# Effect of vertical placement angle on the insertion torque of mini-implants in human alveolar bone

**DOI:** 10.1590/2177-6709.21.5.047-052.oar

**Published:** 2016

**Authors:** Rafael Ribeiro Maya, Célia Regina Maio Pinzan-Vercelino, Julio de Araujo Gurgel

**Affiliations:** 1 MSc in Orthodontics, Universidade Ceuma (UNICEUMA), São Luís, Maranhão, Brazil.; 2 Professor, Universidade Ceuma (UNICEUMA), Masters Program in Dentistry, São Luis, Maranhão, Brazil.

**Keywords:** Anchorage, Torque, Orthodontics

## Abstract

**Objective:**

The aim of the present *ex-vivo* study was to evaluate the effect of the vertical placement angle of mini-implants on primary stability by analyzing maximum insertion torque (MIT).

**Methods:**

Mini-implants were placed in 30 human cadavers, inserted at either a 90° or 60° angle to the buccal surface of the maxillary first molar. Out of 60 self-drilling mini-implants used, half were of the cylindrical type and half were of the conical type. Primary stability was assessed by means of measuring the MIT. Data were subjected to analysis of variance (ANOVA) and Newman-Keuls tests. A significance level of 5% was adopted.

**Results:**

The MIT was higher for both mini-implant types when they were placed at a 90° angle (17.27 and 14.40 Ncm) compared with those placed at a 60° angle (14.13 and 11.40 Ncm).

**Conclusions:**

MIT values were differed according to the vertical mini-implant placement angle in the maxillary posterior area. Regardless of the type of mini-implant used, placement at a 90° angle resulted in a higher MIT.

## RESUMO

**Objetivo:**

o propósito deste estudo foi avaliar a influência do ângulo vertical de inserção dos mini-implantes sobre a estabilidade primária, a partir da análise do torque máximo de inserção (TMI).

**Material e Métodos:**

os mini-implantes foram instalados em 30 cadáveres humanos, em angulação de 90° ou 60° em relação à face vestibular de primeiros molares superiores. Os mini-implantes autoperfurantes (n = 60) utilizados foram 30 do tipo cilíndrico e 30 do tipo cônico. A estabilidade primária foi obtida por meio da quantificação do TMI. Os dados foram submetidos à análise de variância (ANOVA), seguida do teste de Newman-Keuls, considerando-se um nível de significância de 5%.

**Resultados:**

o TMI mostrou-se maior para ambos os tipos de mini-implante na inserção em 90^o^ (17,27 e 14,40 Ncm) em comparação à inclinação de 60^o^ (14,13 e 11,40 Ncm).

**Conclusões:**

o valor do TMI mostrou-se diferente de acordo com o ângulo de inserção vertical na região posterior da maxila. Independentemente do tipo de mini-implante utilizado, a inserção em 90° resultou em valores mais altos de TMI.

## INTRODUCTION

Insertion and removal torques of mini-implants are numerical representations of the quality of primary stability achieved; therefore, such measures are important factors in the success of orthodontic anchorage by means of mini-implants.^1^ Primary stability of mini-implants mainly relies on the device dimension and type, thickness of patient's cortical bone and the insertion technique used.^2^ ^,^ ^3^ ^,^ ^4^ Among several factors related to mini-implant success, cortical bone thickness has been reported as a factor that affects the placement angle.^5^ The current trend is to use mini-implants measuring between 1.4 and 2.0 mm because of the improved primary stability that results from placement into the inter-radicular space, as well as the improved mechanical characteristics of the interface between the mini-implant and the maxillary and mandibular cortical bone.^6^ ^,^ ^7^ Although reducing the placement angle has been proven to increase the contact area between the screw and the cortical bone, angle reductions are not believed to provide greater mini-implant retention.^8^

The recommended insertion technique for mini-implants is placement at an angle relative to the long axis of teeth to help the screw reach the uppermost portion of the alveolar crest, which avoids proximity to the dental roots while placing the mini-implant in an area with more bone contact available because of the conical shape of dental roots. This angle provides adequate mechanical stability without damaging tooth roots.^1, 8-11^

The amount of maximum insertion torque (MIT) represents the quality of primary stability achieved. It is not the only factor related to the success of mini-implants, but it is a measure that can be compared; however, it is important to study the variables that influence MIT. Cortical thickness and age seem to be the patient-related factors that most influence the amount of MIT. For self-tapering mini-implants, MIT has been reported to be between 5 and 10 Ncm. Because partial osseointegration should occur for mini-implants, the MIT value represents not only primary stability, but also the numerical quantification of mini-implant stability.^3^

Technical difficulties in measuring the amount of accumulated stress and cortical bone tissue regeneration in humans has led to different types of *in vitro* studies. Studies of artificial bone, finite elements, animals and cadavers have shown that increased mini-implant placement angle improves stability.^12^ ^-^ ^16^

It is at present unclear how the placement angle influences the MIT.^17^ However, more numerical evidence will help understanding the insertion torque variability for different insertion angles in human cortical bone. 

The aim of the present *ex vivo* study was to evaluate the primary stability of two types of mini-implants (cylindrical and conical) by means of measuring the MIT for their placement at 90° and 60° angulation relative to the buccal surface of the maxillary first molar.

## MATERIAL AND METHODS

A total of 60 self-drilling mini-implants were used with different diameters, but with the same length (Table 1). Thirty of them had a cylindrical body of 1.6 × 9 mm, while the other 30 had a conical body with dimensions of 1.8 × 9 mm. Mini-implants were placed by a single operator in 30 human cadavers (23 males and 7 females) aged between 21 and 39 years old (mean age: 29.4 years), with the posterior maxillary bone and dentition preserved. This study received approval from the institutional review board of UNICEUMA (protocol #2011/0544). The sample was divided into groups according to mini-implant type (1.8-mm conical or 1.6-mm cylindrical) and placement angle, as follows: Group 1, cylindrical mini-implants placed at a 90° angle; Group 2, cylindrical mini-implants placed at a 60° angle; Group 3, conical mini-implants placed at a 90° angle; and Group 4, conical mini-implants placed at a 60° angle (Table 1).

**Table 1:** Mini-implant specifications and codes.

Code	Group	Type	Diameter	Length	Angle	Manufacturer
CL 90	1	Cylindrical	1.6 mm	9 mm	90°	Dewimed (Germany)
CL 60	2	Cylindrical	1.6 mm	9 mm	60°	Dewimed (Germany)
CN 90	3	Conical	1.8 mm	9 mm	90°	Conexão (Brazil)
CN 60	4	Conical	1.8 mm	9 mm	60°	Conexão (Brazil)

**Figure 1:**
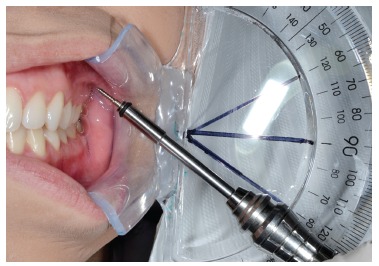
The manual driver tip positioned beside the flat surface of the protractor over the 60° angle.

Because of the split-mouth design of this study, all mini-implants were placed manually on both sides of the maxilla of the same cadaver without pilot drilling. The insertion point was standardised by a millimeter probe used to measure the height at 7 mm from the gingiva margin or the tip of the papilla between the second premolar and maxillary first molar.^18^ MIT values were measured with the aid of a digital torque meter (Lutron TQ-8800, Taipei, Taiwan) and a manual screwdriver suitable for every type of mini-implant. The peak placement torque value obtained during the final turn of the screwdriver during mini-implant placement was recorded for analysis. The 60° and 90° angles were standardised with a protractor positioned laterally to the maxilla. The base of the protractor touched the photograph retractor used to expose the maxillary first molar area. The manual driver tip was positioned beside the flat surface of the protractor over the 60° or 90° lines that run from the protractor base line (Fig 1). Mini-implants of the same type were then placed into the same cadaver at a 90° angle on one side and a 60° angle on the other side.

To evaluate the hypothesis that the vertical placement angle would influence MIT, the values obtained for screws with the same diameter were compared. Two-way fixed-effects analysis of variance (ANOVA) and Newman-Keuls tests were used to compare MIT values. All calculations were performed by means of Statistica software Version 10.0 (StatSoft Inc., Tulsa, OK, USA). A significance level of *p* < 0.05 was adopted.

## RESULTS

The mean MIT values differed among groups and varied between 11.40 and 17.27 Ncm. The mean MIT values for cylindrical mini-implants were 14.13 Ncm for the 60° angle and 17.27 Ncm for the 90° angle. The mean MIT values for the conical mini-implants were 11.40 Ncm for the 60° angle and 14.40 Ncm for the 90° angle (Table 2).

**Table 2:** Means and standard deviation (SD) of MITs (Ncm) for two types of mini-implants and placement angles (n = 15).

Diameter	Angle	Torque (Ncm)	
		Mean	SD
1.6 mm	60°	14.13^b^	3.93
	90°	17.27^a^	3.22
1.8 mm	60°	11.40^c^	1.99
	90°	14.40^b^	2.06

Different superscript letters indicate significant differences between groups.

Evaluating the relationship of MIT values with the placement angles in the axial plane, we observed significant differences between Groups 1 and 2 (*p* = 0.013) and Groups 3 and 4 (*p* = 0.018). Mini-implants placed at 90° in the cortical bone exhibited greater insertion torque than those placed at 60° relative to the cortical bone (Tables 2 and 3).

**Table 3:** Newman-Keuls test for placement angle comparisons for each type of mini-implant.

Comparison	*p* value
(CL 60 *vs*. CL 90)	0.013 *
(CN 60 *vs*. CN 90)	0.018 *

* significant difference (*p* < 0.05).

## DISCUSSION

In order to add information to evaluate the variables that affect mini-implant stability, this research focused on insertion torque to study angle effect as a surgery-related factor for stability. The present study reveals that the vertical placement angle of mini-implants might interfere in the amount of MIT. *Ex vivo* placing of orthodontic mini-implants at a 90° angle resulted in increased MIT. It needs to be emphasized that our results are related to human maxillary alveolar bone, and not to all maxillomandibular areas. For example, for the posterior mandibular area, the vertical placement angle seems necessary to increase the contact area between the screw and the cortical bone.^8^

The literature reports that axial angles from 45° to 70° are the most appropriate for preventing the screw from contacting the dental roots and increasing the amount of bone surrounding the screw.^1^ ^,^ ^9^ However, such angles seem to compromise screw insertion depth and cortical bone integrity, despite providing better contact with the bone of the inter-radicular space compared with greater angles. In other words, it was not related to the 90° insertion angle. Therefore, in our study, the comparison between 90° and 60° angles was proposed because a 60° angle represents a mean point of the rate for vertical angle placement recommended in the literature, which is between 45° to 70°.^1^ ^,^ ^9^ Another study in human alveolar bone did not find any influence regarding placement angle; nevertheless, this clinical study was performed in multiple maxillomandibular areas with three different types of screw.^2^

The placement area between the second premolar and maxillary first molar was chosen because it provides the widest maxillary inter-radicular space and it is, therefore, a safe space for mini-implant placement.^8^ ^,^ ^18^ ^-^ ^22^ The standardization of insertion height at 7 mm away from the interdental papilla made it possible to place mini-implants in the attached gingiva, and also to have a sufficient inter-radicular space.^18^ ^,^ ^19^ ^,^ ^21^ ^,^ ^23^ ^,^ ^24^

Although routinely used in dental studies, human cadavers present some restrictions in clinical applications. There was some concern regarding variability in the post-mortem interval among cadavers; therefore, the experiment used newly deceased cadavers (up to 24 hours post-mortem), so that the cortical bone density of the sample components could be compared.^24, 25^ For greater reliability of the obtained results and to reduce standard deviation, we used more cadavers than the average commonly reported in the literature for this type of study.^14^ ^,^ ^26^ ^-^ ^31^

Similarly to our findings, studies using several experimental models have shown that a mini-implant angle of 90° relative to the cortical bone is advantageous compared with other angles indicated for technical advantages.^13^ Placing orthodontic mini-implants to the alveolar process bone surface at angles less than 90° did not offer force anchorage resistance advantages.^14^

The 1.6-mm screws placed at a 90° angle displayed the greatest insertion torque, suggesting that the increased mini-implant diameter had a significant effect on insertion torque. Greater structural preservation of the cortical bone may have resulted from the lower pressure of the smaller-diameter mini-implant because larger-diameter mini-implants and greater cortical bone thickness require more insertion force.^1^ ^,^ ^9^ ^,^ ^12^

Therefore, variations in screw design and diameter lead to changes in the MIT value.^12^ However, in this study, it was found that 1.6-mm mini-implants exhibited a MIT value higher than 1.8 mm for the same insertion angle. In addition, the commercial brands used in our study had different diameters and types, which did not allow statistical analysis between the types of mini-implant. Nevertheless, we were able to compare the influence of placement angle for mini-implants of the same type. Variation in placement angle may lead to reduced strain on the cortical bone, thus overcoming the increased tendency towards damage associated with increased mini-implant diameter.^13^

The insertion torque values found were similar to those observed in human cadavers ^28^ and in another clinical study.^3^ Thus, the variations found when placement angles were compared represent changes that may also occur in patients. Furthermore, when two brands of mini-implants with different designs and diameters were used, the values differed according to whether they were placed at 60° or 90°. Regardless of the type of mini-implant used, the MIT value was higher when the implants were placed at 90°. This finding means that, for self-drilling mini-implants, placement at a 90° angle should be prioritized to reduce stress on the cortical bone. 

The cylindrical mini-implant (1.6 mm) exhibited a higher MIT value, probably in regard to the cylindrical design of the screws and not as a consequence of the diameter. This reinforces a previous report in the literature, in which conical mini-implants induced more microdamage than the cylindrical ones.^12^ In another study, the range for MIT in human bone was from 5 to 10 Ncm.^3^ The self-drilling mini-implant used in our study exhibited higher MIT values; thus, the high MIT is related to the drill-free system insertion technique.^17^ ^,^ ^32^

Conflicting findings concerning factors that influence MIT values have yielded no evidence to suggest that specific MIT levels result in higher success rates for mini-implants.^17^ Therefore, it is not possible to understand the high torque values obtained here as overload of the cortical bone. Furthermore, our results obtained in human cortical bone will help to provide better association and quantitative records to identify a specific relationship with mini-implant primary stability. In previous human studies, the mini-implant system used increased MIT values with self-drilling insertion when compared with self-tapping.^32^ ^,^ ^33^ Also, variations in the MIT value for human cortical bone have been presented, possibly as a consequence of the different devices (mechanical and digital) used to record torque during mini-implant placement.^34^ In our research, we used a digital instead of mechanical torquimeter to provide more accurate values.^17^ ^,^ ^35^

Histological studies have shown that mini-implant design affects the amount of damage caused to the cortical bone and may be useful in clarifying the types of changes in the area of contact between the mini-implant and the cortical bone associated with MIT.^29^ ^,^ ^36^

The quantity and quality of cortical bone on the failure force of mini-implants have been shown when comparing maxillae and mandibles. In our study, we analyzed the mini-implant/cortical bone interface related to the posterior maxillary alveolar region. The same results should not be extrapolated to other areas, such as the posterior mandibular cortical bone. Cortical bone thickness and bone hardness of mandibles are different when compared with maxillae, mainly in the posterior region.^1^ ^,^ ^7^ ^,^ ^11^

An *in vitro* study, which did not take into consideration different cortical bone thickness, reported that angled insertion provides greater MIT as a consequence of increased contact in the mini-implant-cortical bone interface.^37^ The results of this present study suggest that the characteristics of the alveolar cortical bone should be taken into consideration when determining a suitable placement angle for mini-implant insertion. 

Future studies should analyze whether damage to the cortical bone surface and/or reduced screw insertion depth are associated with the vertical placement angle of the mini-implant. Additionally, further research should be conducted to investigate mini-implant placement in other sites, especially those with different buccal cortical bone thicknesses. 

## CONCLUSION

Based on the results of this *ex vivo* study, MIT values differed according to the vertical mini-implant placement angle in the maxillary posterior area. Regardless of the type of mini-implant used, placement at a 90° angle resulted in higher MITs.
